# Long range recognition and selection in IDPs: the interactions of the C-terminus of p53

**DOI:** 10.1038/srep23750

**Published:** 2016-03-31

**Authors:** Srinivasaraghavan Kannan, David P. Lane, Chandra S. Verma

**Affiliations:** 1 Bioinformatics Institute (A*STAR), 30 Biopolis Street, #07-01 Matrix, Singapore 138671; 2 p53 Laboratory (A*STAR), 8A Biomedical Grove, #06-04/05, Neuros/Immunos, Singapore 138648; 3 School of Biological Sciences, Nanyang Technological University, 60 Nanyang Drive, Singapore 637551; 4 Department of Biological Sciences, National University of Singapore, 14 Science Drive 4, Singapore 117543

## Abstract

The C-terminal domain of p53 is an extensively studied IDP, interacting with different partners through multiple distinct conformations. To explore the interplay between preformed structural elements and intrinsic fluctuations in its folding and binding we combine extensive atomistic equilibrium and non-equilibrium simulations. We find that the free peptide segment rapidly interconverts between ordered and disordered states with significant populations of the conformations that are seen in the complexed states. The underlying global folding-binding landscape points to a synergistic mechanism in which recognition is dictated via long range electrostatic recognition which results in the formation of reactive structures as far away as 10 Å, and binding proceeds with the steering of selected conformations followed by induced folding at the target surface or within a close range.

Proteins generally adopt well-defined tertiary structures under physiological conditions, and this structure largely determines their functions. However there is a class of proteins called intrinsically disordered/unstructured proteins (IDPs/IUPs) that do-not-fold-into well-defined structures and yet are biologically functional^1^,^2^,^3^. These IDPs remain highly conformationally dynamic under native conditions and play important biological roles. IDPs are a large class of proteins. On the basis of their amino acid content and sequences, which is different from that of globular proteins, IDPs are now estimated to represent a significant fraction of many genomes^1^,^4^. For example, roughly three-quarters of the proteins in mammals are predicted to contain disordered segments or are fully disordered. The disorder is thought to result from the reduced contacts among the hydrophobic amino acids. The structural plasticity of IDPs allows them to interact with numerous different targets. These interactions are thought to be mediated by rapid conformational disorder-order transitions^5^,^6^, enabling these regions to interact with a variety of partner proteins with low affinities but high specificities^7^,^8^. Interestingly, although IDPs have low hydrophobic content overall, the motifs they employ to interact are generally short stretches of hydrophobic residues (6 residues)^9^,^10^. The conformational diversity of these interactions has been revealed by numerous structural, biophysical and computational studies^11^,^12^,^13^,^14^,^15^,^16^,^17^.

Experimental and computational studies have revealed two major mechanisms that can be used to describe the binding of IDPs to their targets^11^,^12^,^13^,^14^,^15^,^16^,^17^: conformational selection, resulting from selection of the bound-state-like structure present in the ensemble of uncomplexed IDPs^16^,^17^; induced folding, resulting from an initial encounter between the IDP and the target followed by conformational changes yielding the final shape of the IDP, referred to as the fly casting mechanism^13^,^14^. The link between flexibility, specificity and mechanisms that couple folding and binding of such rapidly interconverting conformations are of great interest. This is further underscored by the finding that IDPs are implicated in diseases such as cancer, neurology and metabolism^15^,^18^,^19^. With increasing attention being paid to the disruption of protein-protein interactions in therapy this assumes an even greater importance. While it may appear that the structural heterogeneity of these regions makes them unlikely targets of small molecules, two recent reports have opened new windows of opportunity: the exploitation of entropic expansion of IDPs with small molecules^20^ and the discovery that a tin(IV) oxochloride cluster selectively targeted a disordered region of the TFIID transcription complex^21^.

p53 is a transcription factor consisting of ordered and disordered regulatory regions engaged in multiple interactions, and is one of the most extensively studied IDPs^22^,^23^. The p53 protein consists of 393 residues and can be divided into three functional regions: (i) an N-terminal domain (residues 1–93) containing a transcriptional activation domain and a proline-rich domain; (ii) a core DNA-binding globular domain (residues 102–292), which contains most of the mutations found in cancers; and (iii) a C-terminal domain (CTD) consisting of a tetramerization domain (residues 320–356) and a regulatory domain (residues 363–393). The extreme CTD is reported to regulate specific DNA-binding activity of p53^24^, either by altering the conformation of p53 or by interfering sterically with the ability of the protein to bind DNA^24^,^25^,^26^. Deletion of this regulatory region, binding of antibodies, post-translational modifications including phosphorylation and acetylation abolish this effect^25^,^26^. The CTD has also been reported to modulate the stability and cellular localization of p53^27^.

Given the diversity of functional interactions of the p53CTD, we decided to investigate its conformational dynamics in the context of the structural information available. The crystal structures of the p53CTD bound to five different globular proteins, adopting four different conformations^28^,^29^,^30^,^31^,^32^ is available (Fig. 1). The p53CTD adopts an α-helix (residues 377–388) when bound to S100 calcium-binding protein B (S100B(ββ)), (PDB 1DT7^28^); a *β*-strand (residues 379–387) when bound to Sirtuin, (PDB 1MA3^29^); a *β*-turn (residues 380–386) when bound to cAMP response element-binding (CREB) binding protein CBP bromodomain, (PDB 1JSP^30^); no secondary structure (residues 369–374) when bound to the histone methyltransferase Set9 (PDB 1XQH^31^) or (residues 378–386) when bound to the cyclin A/cyclin-dependent protein kinase 2 complex (PDB 1H26^32^). These complexes reveal a diversity of interactions: mostly hydrophobic when bound to S100B(ββ) or to CBP, a combination of hydrophobic and hydrogen bonds (hbonds) when bound to Cyclin A and electrostatics complemented by hbonds when complexed to Sirtuin or to Set9. Overlapping regions of the CTD (sequence _363_RAHSSHLKSKKGQSTSRHKKLMFKTEGPDSD_393_ Fig. S1) are stabilized in different binding pockets through such diverse interactions, emphasizing the inherent plasticity of this disordered region. Given the paucity of experimental data on the details of how these interactions are mediated at the atomic level, we address this issue using molecular simulations in this current study. Molecular simulations have been used in earlier studies to interrogate the p53CTD-protein complexes, providing valuable insights^33^,^34^,^35^,^36^: p53CTD adopts transient α-helices in solution^34^,^35^; the binding of p53CTD to S100B(ββ) induces secondary structure in p53 through a fly casting like process^34^. We now build upon this work by exploring the binding between the p53CTD and five of its partners using detailed atomistic simulations. We explore the binding process using a two-pronged strategy: we first use steered molecular dynamics simulations (SMD) to examine the process of unbinding of the peptide from its complexed state and further complement this with approximating the process of binding, whereby we place the peptide in different conformations at different distances from the binding pocket and carry out umbrella sampling molecular dynamics (USMD) simulations. The stability of the complexes is examined using a series of standard molecular dynamics (sMD) simulations. The conformational space sampled by the peptide is explored using replica exchange molecular dynamics (REMD) simulations (and are outlined in the supplementary material). The five binding partners chosen for this study represent diversity in size, function, and nature of interactions with the p53CTD, and crucially, are available in crystallographically/NMR resolved complexes.

## Materials and Methods

### System preparation

The experimental structures of five p53CTD – receptor complexes were obtained from the protein data bank: (a) For the p53CTD-S100B(ββ) complex, the first structure of the NMR ensemble^28^ was chosen; in the NMR models, S100B(ββ) exists as homodimer with a 22-mer fragment (residues 367–388) of p53CTD bound to each unit of S100B(ββ) but for simplicity, only one monomer complex was used in the current study. Only a short fragment (residue 377–388) of p53CTD was used as the rest of the peptide is disordered, highly flexible and doesn’t interact with the S100B(ββ) in any of the NMR models. Two calcium ions that were bound to S100B(ββ) were retained. (b) In the case of the p53CTD–Sirtuin complex, a 9-mer fragment (residue 379–387) of p53CTD co-crystallized with Sir2 was used^29^. Lys382 of p53CTD was acetylated as seen in the original structure. A zinc ion coordinated by Sir2 was retained. (c) The crystal structure of p53CTD-Cyclin A complex contains a 9-mer (residue 378–386) p53CTD bound to a complex of cyclin A and phosphorylated cyclin-dependent protein kinase 2 (cPDK2)^32^. Since the p53CTD only interacts with cyclin A, cPDK2 was not included. Only one copy of the complex from the crystallographic asymmetric unit is considered here. (d) In the NMR models of p53CTD–CBP bromodomain, a 20-mer fragment of p53CTD is complexed with the CBP bromodomain^30^. However only a short fragment (residues 380–386) of p53CTD is bound to the receptor protein and the rest of the peptide is disordered, highly flexible in all the NMR models and doesn’t interact with the CBP bromodomain. Therefore only a shorter (residues 380–386) fragment of p53CTD was used in the p53CTD-CBP complex and the first model of the NMR ensemble was chosen for the current study; Lys382 of p53CTD is monoacetylated as is necessary for binding. (e) The crystal structure of p53CTD-methyltransferase (Set9) complex contains a 6-mer fragment (residue 369–374) of the p53CTD peptide^31^. Although the crystallographic asymmetric unit contained two copies of the complex, only one was considered here. Lys372 of the p53CTD was monomethylated as it is in the original structure. For simulations starting from fully extended conformations of p53CTD, an extended p53CTD (residue 370–389) structure was generated using the Xleap module of the Amber11 package^37^.

### MD simulations

The five p53CTD–receptor structures chosen above were used as starting structures for the sMD simulations of the complexes, and for the apo p53CTD and receptor simulations corresponding structures extracted from the complexes were used. In each structure the N- and C- termini of the p53CTD fragment was capped with acetyl and amide groups respectively. Hydrogen atoms were added to the experimental structures using the Xleap module of the Amber11 package. All the systems were neutralized by the addition of counter ions. The neutralized systems were solvated with TIP3P^38^ water molecules to form a truncated octahedral box with at least 10 Å separating the solute atoms and the edges of the box. MD simulations were carried out with the Sander module of the Amber11 package in combination with the parm03 force field^39^. Force field parameters for acetylated, monoacetylated Lysine and monomethylated Lysine were used as described elsewhere^40^. All the systems were first subjected to 1000 steps of energy minimization. This was followed by MD simulations, for which the protein was initially harmonically restrained (25 kcal mol^−1^ Å^2^) to the energy minimized coordinates, and the system was heated up to 300 K in steps of 100 K followed by gradual removal of the positional restraints and a 1 ns unrestrained equilibration at 300 K was carried out. The resulting system was used for the MD simulations. A total of 45 (5 complexes, 5 receptors, 5 peptides in triplicates) MD simulations were carried out for 100 ns each, totaling 4.5 μs (Table S1A).

### Steered Molecular Dynamics

To investigate in some detail the process of (un)binding of the peptides to their receptors, the equilibrated structures of the five p53CTD–receptor complexes were subjected to SMD simulations. SMD is a biasing method that utilizes time-dependent external forces to induce structural changes in biomolecules^41^,^42^. SMD is rooted in single molecule pulling experiments, and forces the system to evolve away from its initial equilibrium condition, thus accelerating the transitions between different energy minima. In recent years SMD has become very popular and has been extensively applied to several biological processes, including folding/unfolding, transport of ions and organic compounds through membrane channels, and has provided insights into the ligand (un)binding pathways/mechanisms^43^,^44^,^45^. In the current work, to facilitate peptide unbinding, a set of distance restraints between receptor and p53CTD peptide atoms that are derived from the crystal structures of the p53CTD–receptor complex were used as the pulling variable during SMD. A spring constant (5 kcal/mol/Å^2^) and pulling velocity (0.00005 Å/timestep ) were chosen as pulling parameters to prevent distortions to the receptor as a consequence of pulling. For each model, five independent SMD simulations, all starting with the same structure but different initial velocities, were carried out for 10 ns each (Table S1C) using the collective variables (colvar) module in NAMD^46^.

### Umbrella Sampling

To obtain a detailed understanding of the free energies along the pathways of (un)binding of the p53CTD and their binding partners, USMD simulations were carried out, with the peptide placed at different distances from its binding site/pocket and restrained by an external harmonic force. The distance restraint involved the centers of mass of the binding site atoms of each protein and peptide and were imposed using the collective variables (colvar) module in NAMD^46^ with a force constant of 50 kcal/mol. USMD was carried out with the p53CTD in its bound conformation placed at the binding site as well as at distances of 10 Å, 20 Å and 30 Å from the binding pockets, with 25 ns simulations carried out at each distance. Similarly for each binding partner, the simulations were repeated with the p53CTD in its non-native bound conformations at the binding pocket as well at the three different distances (Table S1D). Only three of the five p53CTD–receptor complexes (p53CTD-S100B(ββ), p53CTD-Cyclin A, p53CTD-Sirtuin) were chosen for the USMD as the bound p53CTD (approximately the same length) in these three cases represent both the ordered (different secondary structures) and disordered states. The other two systems (p53CTD–CBP, p53CTD-Set9) were not considered here because (a) the region of p53CTD co-crystallized with Set9 is very different from the others and (b) the p53CTD binding site in the CBP is very narrow and replacement of the native bound p53CTD with nonnative bound peptide resulted in huge steric clashes. In all the three USMD simulations, the same region of p53CTD was used (_378_SRHKKLMFK_386_).

### Binding Energy calculations

Molecular Mechanics Generalized Born Surface Area (MMGBSA) and Poisson Boltzmann Surface Area (MMPBSA) methods were used for the calculation of binding free energies between the peptides and their partner proteins^47^. The molecular mechanics energy term used in these calculations represents the internal bonded energy (energy of bonds + angles +dihedrals) as well as the non-bonded van der Waals and Coulomb energies. Two sets of MMPBSA/MMGBSA calculations were carried out for each system (complexed state and apo states). For the calculation of binding energies from the complex and apo simulations the last 50 ns of the sMD trajectories were used. For the USMD simulations, snapshots from the last 15 ns of USMD trajectories of both the native and non-native complexes with the peptide placed at different distances were used for binding energy calculations.

### Analysis

RMSD, RMSF and secondary structure calculations were all carried out with the PTRAJ module in AMBER. Native binding contacts are defined as contacts between protein and peptide residues in their crystal structures when their Cα atom are closer than 6.5 Å. Native binding contacts at 10 Å are also calculated similarly by moving the peptide 10 Å away from the centre of mass (COM) of the binding site in its corresponding experimental structure. Cluster analysis was based on the pair-wise cartesian root mean squared deviation of only the heavy atoms between conformations, with an rmsd cutoff of 2 Å and using the kclust program in the MMTSB-tools^48^. The VMD (Visual molecular dynamics) program^49^ and Pymol^50^ was used for visualization of trajectories and preparation of figures.

## Results

**Figure 2:**
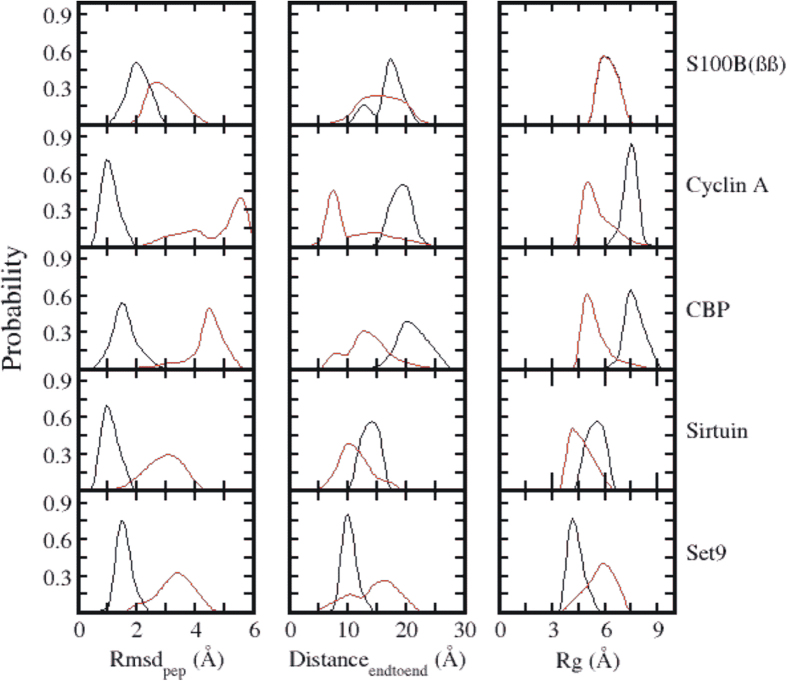
Probability distributions of RMSD, end to end distances and Rg of the structural ensemble of five different but overlapping p53CTD peptide from their bound state, sampled during MD simulations in the presence (black) and absence (red) of their binding partners.

Standard MD simulations (multiple copies) of the complexes show that the simulations are stable and the peptides remain close to their bound states (rmsd < 2.5 Å, Fig. 2). In contrast the simulations of the peptides in their apo states (either starting from the bound states or from a fully extended state) shows that the peptides are highly flexible and adopt multiple conformational states (rmsd > 4 Å, Figs 2 and S2). The apo peptides adopt collapsed conformations (Figs 2 and S2) and sample the bound state conformations with varying probabilities (Figs 2, S3 and S4).

### Coupled binding and folding from unbinding simulations

Simulations of the peptides in their apo states show that the peptides adopt the bound state conformations and also collapsed conformations (Figs 2, S3 and S4). Hence to examine whether binding occurs through conformational selection, induced fitting or some combination, we need to explore the actual process of binding of the peptide. We first study the technically easier process of unbinding using SMD. A key assumption is that binding/folding is largely a reverse of unbinding/unfolding.

**Figure 3:**
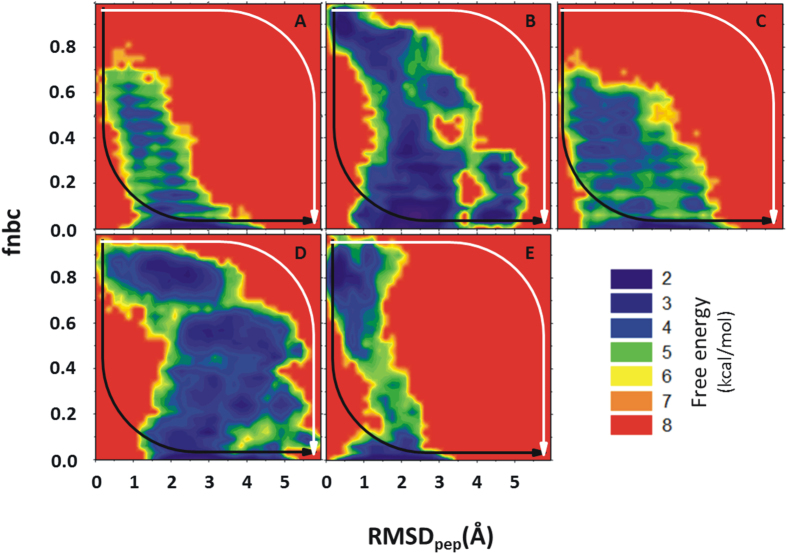
2D free energy landscape as a function of fractions of native binding contacts (fnbc) formed between the receptor – p53CTD peptide fragment and rmsd of peptide (rmsd) from the peptide – receptor complex. SMD simulations were carried out for unbinding of p53CTD bound with five different receptors (**A**: S100B(ββ), **B**: Cyclin A, **C**: CBP, **D**: Sirtuin **E**: Set9). All the conformations sampled during SMD (5 independent for each system) are included in the 2D free energy calculations. The two paths describe the conformational selection (black) and induced fold (white) mechanisms.

To monitor the conformational flexibility of the p53CTD peptide during pulling, peptide rmsd was measured relative to its starting experimental bound structures. Changes in receptor–peptide interactions upon peptide unbinding were monitored by recording the fraction of native binding contacts (fnbc), together with the distance between the center of mass (COM) of the binding site and the peptide fragment. Although with increasing COM, an increase in peptide rmsd and a decrease in the fnbc was observed during SMD, interesting trends were observed. In the case of S100B(ββ)-p53CTD and CBP-p53CTD complexes, where the complex is stabilized mainly by hydrophobic interactions, rapid loss in fnbc was already observed (>60%) at COM of ~10 Å (Fig. S7). In other cases where the bound conformation of the peptide is stabilized by numerous h–bond interactions (Sir2, Cyclin A and Set9 complexed with p53CTD) a lag in reduction of fnbc was observed, with more than 50% of the native contacts existing even at COM of ~15 Å. Overall, the changes in peptide conformations appear to follow a common trend, with a rapid increase in rmsd (~3 Å) (Fig. S8) for all the peptides by ~10 Å from the binding site. However further changes in the peptide conformations appear to be less rapid, with the conformations of the bound states such as helix or sheet existing partially even at distances of ~20 Å from the receptor. The disordered bound conformations show rapid fluctuations and become partially ordered. The 2 D free-energy landscape (Fig. 3) revealed varied populations in several minima for the different complexes. A minima at the upper left corner of each graph corresponds to the complexed state (with the peptide in its folded/bound state with rmsd < 1.5 Å and fnbc >~60%) for the complexes of p53CTD with Cyclin A, Sirtuin and Set9, all of which are characterized by a number of hydrogen bonds mediating interactions between the proteins and the peptides. Such a native minima is less populated and slightly shifted (rmsd < 1.5 Å and fnbc ~40–60%) for the complexes between p53CTD with S100B(ββ) and p53CTD with CBP, where the protein-peptide interactions are governed mainly by hydrophobic interactions. The energy landscapes are also decorated with other minima that correspond to partially unfolded and unbound states, with rmsd >3.0 Å or fnbc <40%. There are several sparsely populated minima that are separated by low energy barriers that are rarely/transiently populated. Comparison of the landscape of all the five combined SMD simulations for each system shows that in all the simulated systems the bound/folded peptide follows two different pathways to reach its unfolded/unbound conformation. Along path1 (white arrow Fig. 3), the pc53CTD fragment rapidly loses its bound conformation (rmsd > 3 Å) but retains most of the native binding contacts (fnbc >60%), suggesting that the peptide becomes disordered upon exiting from the binding pocket. This suggests that order must be induced in the peptide by the receptor upon binding, and is seen for the p53CTD complexes with Sirtuin and Cyclin A - this is the induced folding mechanism^13^,^14^,^51^. Along path2 (Black arrows Fig. 3), the bound conformation (alpha helix or beta sheet or disordered state) is retained (rmsd < 2.5 Å) or at least partially retained, with more than 70% of the fnbc lost already. This suggests that the peptide adopts the bound conformation even before it binds to its partner, and the native binding contacts play a role in selecting such a folded/bound conformation of the peptide, a scenario referred to as the conformational selection mechanism^16^,^17^ and appears to characterize complex formation between p53CTD and S100B(ββ), CBP or Set9.

In contrast to the increased conformational fluctuations/changes of p53CTD peptides, all the corresponding receptor conformations except two remained relatively stable (rmsd < 3 Å, Fig. S8) upon pulling the peptide from its binding pocket. Increased receptor fluctuations were observed for S100B(ββ) and CBP proteins. In the case of S100B(ββ) it is known that the protein undergoes conformational changes upon peptide binding [33]. The p53CTD binding site in CBP is made up mostly by loops and it is clear that the binding of the peptide stabilizes the loop conformations.

### p53CTD–receptor initial encounter and complex formation

The SMD yields a trajectory of the conformational changes associated with the process of unbinding. However it has its limitations, particularly at larger distances of the peptide from the binding site, where the sampling is insufficient. To overcome this and explore the conformational landscapes of the peptides in detail, we position the peptides at specific distances (0, 10, 20 and 30 Å) from the binding site/pocket and restrain them by an external harmonic force and subject the protein - peptide complexes to USMD (each peptide was placed at each location in 3 different conformations as outlined in Methods).

**Figure 4:**
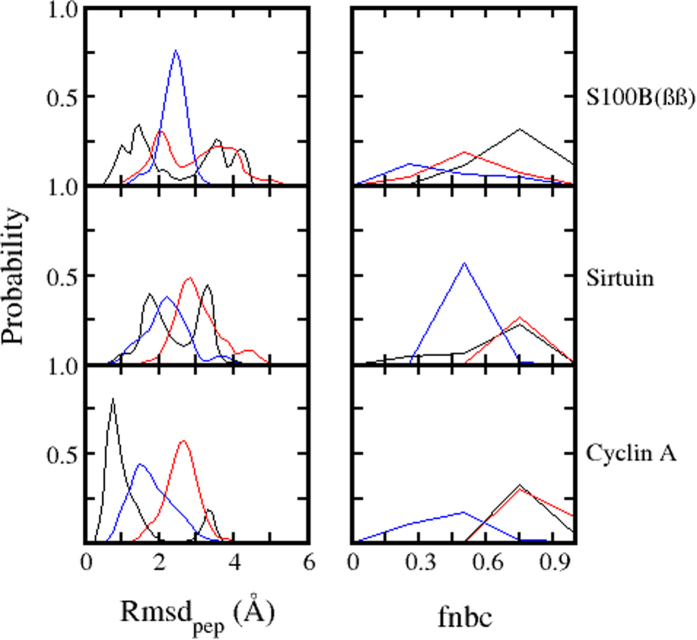
Distributions of the rmsd of the p53CTD peptide (Alpha helix: black, Beta sheet: red, disordered: blue) taken from the p53CTD – receptor complex and of the fractions of native binding contacts (fnbc) formed between the receptor and the p53CTD peptide fragment, when the peptide is placed 10 Å away from the binding site.

Irrespective of the conformation used (native or non-native), when the peptides are placed at larger distances (20 Å and 30 Å) from the binding sites, their dynamics are characterized by increased flexibility and rapid unfolding and refolding, suggesting that the receptors do not influence their behaviour. However, at 10 Å, the corresponding native bound (alpha helical, beta sheet and disordered) conformations of the p53CTD fluctuated significantly with some unfolding (Fig. 4) yet significantly populated in their corresponding native folded states with the associated native binding contacts (fnbc ~60%). When the native bound conformations of the peptides are replaced by the native conformations seen in the other 2 complexes, folding and unfolding of secondary structures are seen and surprisingly, partial formation of native bound conformations (alpha helix in the case of S100B(ββ) and beta sheet in the case of Sir2) are also observed (Fig. 4). This clearly reflects the long range influence of the receptor on modulating the conformational dynamics of the peptides; native and non-native contacts govern these dynamics.

**Figure 5:**
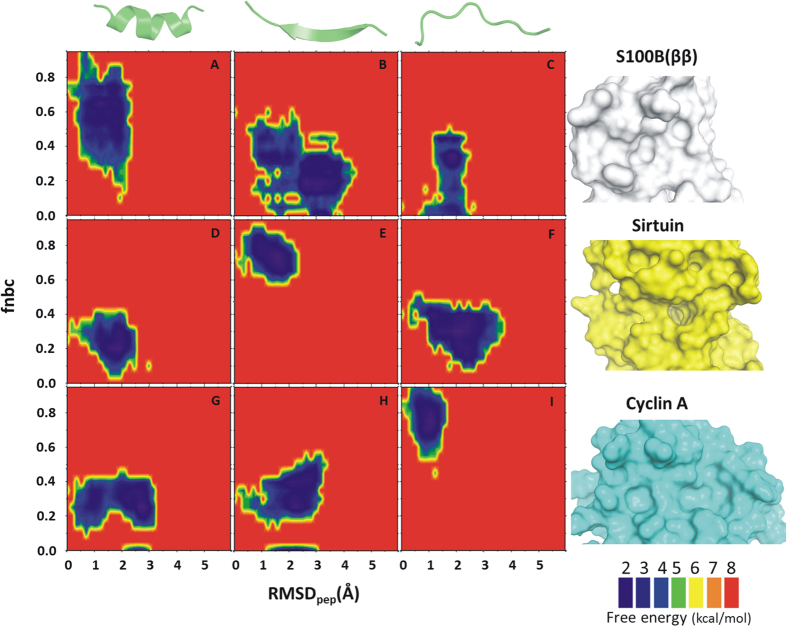
2D free energy landscape as a function of fractions of native binding contacts (fnbc) formed between the receptor – p53CTD peptide fragment and rmsd of peptide (rmsd) from the peptide – receptor complex. All the conformations sampled during USMD simulations with bound form of p53CTD fragment either in its native (**A,E,I**) or non-native (**B**–**D,F**–**H**) conformations bound with three receptor proteins – S100B(ββ) (**A**–**C**), Cyclin A (**G–I**) and Sirtuin (**D**–**F**) at the binding sites are included in the 2D free energy calculations.

When the peptides are placed in the binding sites in their experimentally bound states, the simulations were stable as expected and no major conformational changes were observed. The 2D energy landscape of the fnbc relative to the rmsd of the peptides shows a single minima with rmsd < 2 Å and fnbc >70% for all the three native complex simulations (Fig. 5). However, when the peptides are placed in the binding sites in their non-native conformations, unsurprisingly, increased fluctuations were observed at the binding interface. For example in the case of the S100B(ββ) protein, where the binding site is highly hydrophobic, when the alpha helical conformation of p53CTD was replaced by a beta sheet or a disordered conformation, increased fluctuations were observed for both the peptide as well as S100B(ββ), especially in the binding site residues. Despite the binding site of S100B(ββ) protein being large and highly hydrophobic and highly suitable for the binding of an alpha helix, when the peptides were placed in non-helical states, they could not be induced into the alpha helical states (Fig. 5); this of course could also result from insufficient sampling. Although the binding pocket is hydrophobic, there are charged residues on both sides of the binding pocket and these residues interact with complementary charged residues of p53CTD, and such non-native contacts appear to limit the formation of the alpha helical state. Similar trends were observed for the other two proteins, Sir2 and Cyclin A, when the non-native conformation of the peptide was located at the binding interface (Fig. 5). When simulations were carried out with the peptide bound in a non-native alpha helical conformation, increased fluctuations and partial unfolding was observed with an rmsd of ~3 Å. As both these enzyme either exhibit a narrow and slightly charged binding interface (in the case of Sir2), which is not suitable for the binding of alpha helices (due to the absence of stabilizing hydrophobic interactions in the case of Sir2), increased fluctuations as well significant absence of native binding contacts were observed. However both Sir2 and cyclin A tolerated disordered non-native conformations of the peptides as non-native h-bond interactions with the binding site residues were sufficient to stabilize the peptides. But, in none of the cases, formation of native bound conformation or native binding contacts was observed when the simulations were initiated from non-native bound conformations. This contrasts with the induced folding mechanism, where the peptide binds before it folds. While it is possible that longer simulation times may witness such transitions, analysis of data across different timescales in our simulations shows convergence (Fig. S9).

**Figure 6:**
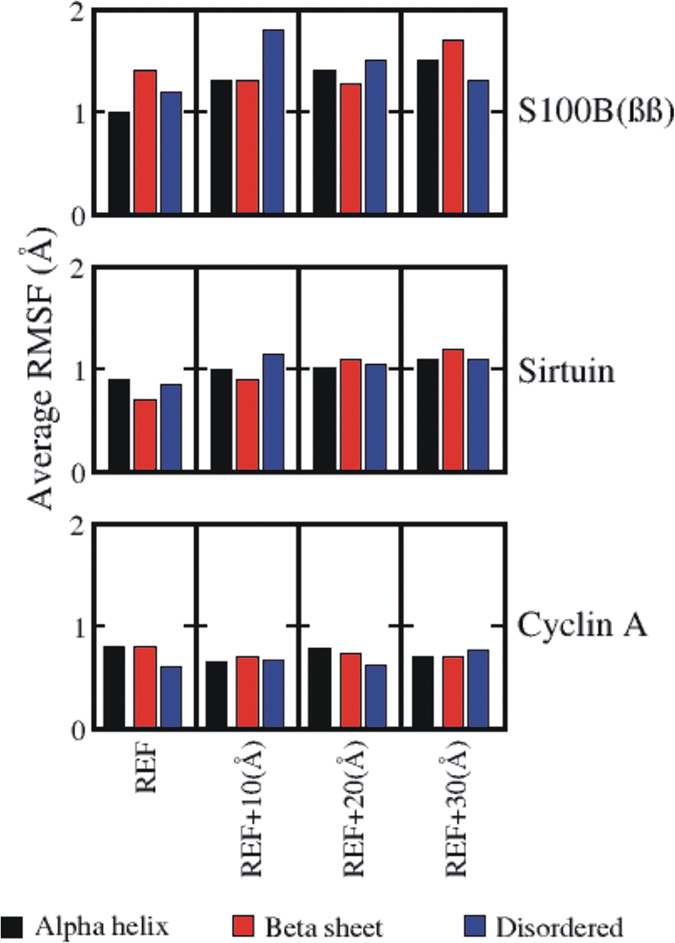
Average rmsf of only the binding site residues of the structural ensemble of three different p53CTD binding receptors, with the peptide in its native and non-native conformation bound with the receptor at the binding site and at various distances from the binding site.

Although the receptors remain stable overall, irrespective of the bound conformations of the peptides, and of their separation from the peptides (10, 20 and 30 Å), varying patterns of fluctuations of residues in and around the binding sites were observed. When the peptide is bound, the binding pocket residues do not fluctuate much, and if the peptide is in its native conformation, then the fluctuations of the binding site residues are the lowest (Fig. 6). However surprisingly, these fluctuations increase when the peptide is between 10 and 20 Å after which they get attenuated. This pattern is observed for residues in the binding pocket that either make direct contacts with the peptide in its bound state or are not specific, thus suggesting that recognition begins to occur at distances between 10–20 Å.

**Figure 7:**
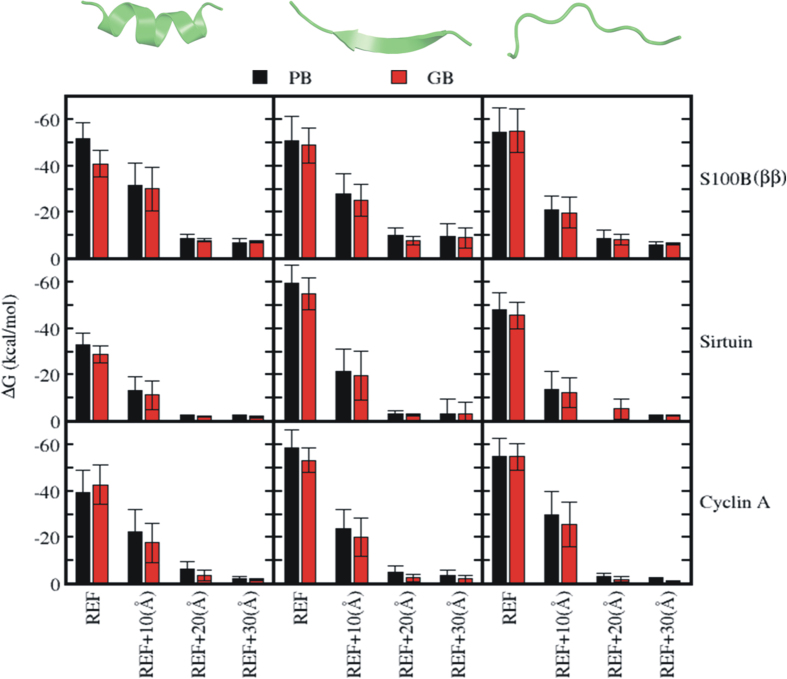
Binding free energy calculations of three p53CTD peptide – receptor complexes calculated with MMPBSA and MMGBSA. All the conformations sampled during USMD simulations with bound form p53CTD fragment both in its native and non-native conformations at the binding site and various distances from the binding site are included in the binding energy calculations.

**Figure 8:**
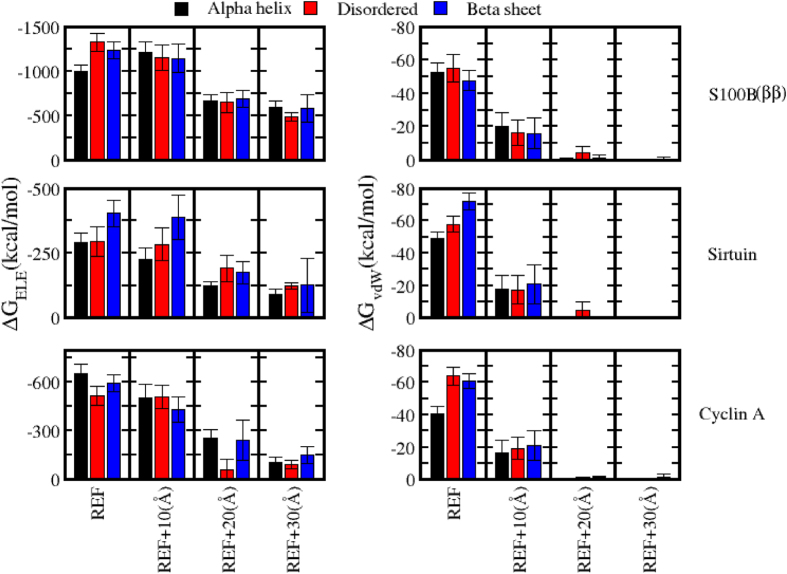
Contribution of Electrostatics and Van der Waals to the total binding free energy of three p53CTD peptide – receptor complexes. All the conformations sampled during USMD simulations with bound form p53CTD fragment either in its native and non-native conformations at the binding site and various distances from the binding site are included in the binding energy calculations.

To characterize the energetics associated with the process of coupled binding and folding, we carried out MMPBSA and MMGBSA type binding energy calculations on the ensembles, generated by the USMD simulations. In all the simulated complexes, when the peptide was positioned at distances far from the binding site (20 Å and 30 Å), irrespective of the conformation that the peptide adopts, the total binding energy was low suggesting little influence of the two molecules on each other (Fig. 7). However at a distance of 10 Å from the binding sites, the total binding energy favored the association/complexation process in all the simulations (Fig. 7). Again at the binding interface, the formation of receptor–peptide complex is highly energetically favored, with the native complexes clearly favored. Interestingly such a clear preference for the native conformations of the peptides was apparent even at 10 Å (Fig. 7). Both the electrostatics and van der Waals components begin to contribute at 10 Å from the binding site (Fig. 8) although, as expected, the long range component i.e. the electrostatics dominated (however when we factor in the desolvation penalties (Fig. S10), the magnitude of electrostatic contributions is attenuated); and this component was strongest only when the peptide was in its native conformation. At the binding site, the complexation is energetically more favored with the native bound conformation of the peptide with both electrostatics and VdW making up the binding energy; as expected, the contribution of VdW is much more than it is at 10 Å. This is not surprising since the binding is accompanied by conformational rearrangements for better/tight packing and stabilization of the complex structure. Our USMD simulations of the receptor–peptide complexation suggest that each receptor preferentially binds to p53CTD in which structural elements that resemble the bound state exist, ie, through an extended conformational selection mechanism. A point to note is that our electrostatic interactions were evaluated without including the effects of salt; however estimates of the salt effects (data not shown) show that the patterns of changes across the systems remain similar to the no-salt case; the interactions are attenuated only slightly.

To test the above hypotheses, we next decided to carry out in silico mutagenesis studies. We chose the S100B(ββ) and Cyclin A systems since both receptors exhibit a wide hydrophobic cavity (partially charged in the case of Cyclin A), yet p53CTD binds S100B(ββ) as a helix while it binds Cyclin A in an extended disordered conformation. We identified Lys381 and Phe385 from p53CTD as contributing the most to the binding with Cyclin A (Fig. S11) and therefore mutated them to Ala. The double mutant p53CTD^K381A,F385A^ shows increased helicity as compared to the wild type in solution, although the simulations were initiated from both, the helical (as in the complex with S100B(ββ) as well as disordered (as in the complex with Cyclin A) states. A loss in the binding energy to Cyclin A (ΔΔG_WT-MUT_ = +9.8 kcal/mol) was seen; wildtype p53CTD binds to Cyclin A in the disordered state and increased helicity disfavors the binding. In contrast, a slight increase in the binding energy was observed for binding to S100B(ββ) (ΔΔG_WT-MUT_ = −2.3 kcal/mol). Once again, these effects were pronounced already at ~10 Å from the binding site, with increase in binding energy to S100B(ββ) (ΔΔG_WT-MUT_ = −4.8 kcal/mol) and decrease in binding energy to Cyclin A ((ΔΔG_WT-MUT_ = +7.4 kcal/mol). Our simulation suggests that increasing the population of the ordered state (in this case the alpha helical) results in improved binding when conformational selection operates (as it appears to occur in the case of p53CTD binding to S100B(ββ)). However such an increase in the ordered state results in a reduction in the intrinsic flexibility of the p53CTD and therefore disfavors the binding when induced folding operates (as it appears to occur in the case of binding to Cyclin A).

## Discussions

A major characteristic of IDPs is their ability to fluctuate among several alternative conformations. This enables them to easily bind to multiple receptors. However this makes understanding mechanisms of their specificity difficult. In this context MD simulations have been increasingly used to detail their folding and binding. One such IDP, the C-terminal domain of the tumour suppressor protein p53, interacts with several proteins^28^,^29^,^30^,^31^,^32^. In the current work we have combined equilibrium and non-equilibrium atomistic explicit solvent MD simulations to investigate the binding of this IDP with a subset of its binding of partners which include S100B(ββ), Cyclin A, CBP, Sirtuin and Set9.

MD simulations reveal that the free p53CTD peptide is quite flexible in its apo state adopting multiple conformations including those observed in the experimentally resolved complexes. A particular stretch of the sequence tends to be more ordered compared to the rest of the p53CTD; this is the region that is common in binding to the various partners. Recent observations have demonstrated that such persistent residual structure in disordered ensembles have important roles in initial recognition and binding^52^,^53^. Existence of such folded-like/bound-like conformations hints at the existence of the conformational selection mechanism in recognition/binding. However recent NMR and coarse grained molecular simulations on other protein–peptide interactions suggest that increasing the amount of structure in the unbound states can lead to attenuations in the binding rates^54^,^55^,^56^.

To study the process of recognition and complexation, we first pull the peptides slowly out of their binding pockets. We see that the peptides lose their order and become disordered/unfolded. They sample a wide variety of conformations, including the folded/bound conformation, even at large distances from their target proteins. This transition can occur along two distinct pathways: (a) along path1 the rate of unfolding (ordered to disordered transitions) is faster than the rate of unbinding (loss of binding contacts). (b) along path2, the rate of peptide unbinding is faster than of its unfolding, and the peptide still retains its folded/bound conformation despite losing most of its native binding contacts.

Thus, the free peptide simulations support the conformational selection mechanism, but the peptide unbinding simulations suggest that both conformational selection and induced folding mechanisms are employed depending upon the receptor. These simulations also indicate that the ordered/structured state of p53CTD is less stable in the absence of its binding partners. The peptide rapidly interconverts between several sub-states that are probably separated by small free energy barriers. These conformational fluctuations also likely contribute to efficient IDP recognition by allowing the peptide to fold rapidly upon binding through fly-casting^57^. However in recent years the magnitude of this effect has been questioned, with suggestions that it may at most result in ~1.6 fold acceleration^57^,^58^,^59^. Indeed, this mechanism was also based on the assumption that unstructured regions/peptides adopt extended conformations which in turn increase the capture radii, thus resulting in higher k_on_. But recent studies have shown that the unbound IDPs tend to be much more compact than previously assumed (as is also seen in our simulations), further reducing the proposed fly-casting affects^57^,^58^,^59^.

We additionally simulate the initial encounter during complex formation by placing the peptides at increasing distances and in varying conformational states from the receptors. We find a marked attenuation in flexibility (rapid folding and refolding) of the peptide as the peptide approaches its binding partner concomitant with the formation of contacts with the receptor proteins. While only the native complex is favoured at the binding site, we also see structural and energetic (largely electrostatic) bias towards the native conformations of the peptides even at separations of ~10–15 Å. It appears that electrostatic interactions between charges on IDPs and those near the binding sites are responsible for the long range steering as well as catalyzing efficient folding after the initial encounter. This underpins the regulations of these interactions by post-translational modifications that add or remove charges on IDPs. After the initial steering, the initial contacts facilitate the folding of peptide on the receptor surface and finally the packing (van der Waals interactions) between the receptor and peptide stabilizes the ordered form of the peptide. Thus the driving force required for coupled folding and binding is provided by favorable interactions between the peptide and the receptor and reduced entropic penalty which results from the binding of pre-formed structural elements.

The above mechanisms were further examined by simulations of a postulated double mutation which suggests that increasing the population of the ordered state results in a reduction in the intrinsic flexibility of the p53CTD. In the case of S100B(ββ) which binds an ordered helical state of p53CTD (conformational selection), this increased order resulted in an improvement in affinity. In contrast, the binding to Cyclin A, which prefers a disordered conformation (induced fit), binding was reduced. A similar observation has been made for the E3 rRNase domain-Im3 interactions^60^, where an increase in disorder brought about by a mutation in E3 rRNase, resulted in reduced binding.

Our studies suggest that for the complexes mediated by van der Waals interactions, conformational selection appears to dominate the process of recognition, whereas for the complexes where h-bonds mediate interactions, induced folding appears to be the dominant mode of recognition. Similar features have been reported for complexation involving proteins with ligands and with DNA^61^. The authors of this study show how systems that are electrostatically driven, such as negatively charged DNA and positively charged regions of DNA binding proteins, are likely dominated by induced fit mechanisms. Similarly, we find that the interactions between positively charged p53CTD and the negatively charged Sir2 are dominated by induced fit. In contrast, the interactions between the largely hydrophobic S100B(ββ) and positively charged p53CTD, characterized by short range interactions are dominated by conformational selection mechanisms. Based on our simulation results we propose that p53CTD binding occurs synergistically via a combination of preformed structural elements (conformational selection) and coupled folding and binding (induced folding). Of course, both mechanisms must operate synergistically, with conformational selection being important for the initial recognition followed by the reactive complex relaxing into the final bound state through multiple induced folding processes. Similar synergism has also been proposed for the binding of various IDPs to their targets. These include interactions between the c-Myb and the KIX domain^62^ and between the transactivation domain binding of nuclear co-activator binding domain (NCBD) and the transcription coactivator CBP activation domain of p160 steroid receptor coactivator (ACTR)^63^. Although current evidence appears to suggest one or the other mechanism governs the interactions between the several IDP-protein interactions such as phosphorylated kinase-inducible domain (pKID) of the cAMP-response element binding (CREB) protein and the KIX domain of the transcriptional coactivator CREB-binding protein (CBP)^64^,^65^, we hypothesize that similar synergism will characterize these interactions. Of course IDPs vary greatly in their conformational propensities, thereby resulting in mechanisms that combine the two processes to different extents. This manifests at one extreme in the KIX domain employing these two mechanisms to different degrees in interacting distinctly with two different IDPs (pKID^64^ and TAD of c-Myb^62^). In contrast, we find in our study, that the same IDP utilizes these mechanisms to varying degrees as it flirts with different receptors, each resulting in distinct biological outcomes.

Implications for oncogenic mutations: What implications might this have in biology given that p53 is highly mutated in cancers? Analysis of the mutations deposited in the (a) IARC database^66^ and in the (b) COSMIC database^67^ of both the N- and C-termini of p53 show low mutability, similar to that reported for PTEN^68^; in contrast there is some evidence that missense mutations in disordered regions do have a functional impact in diseases^69^. Do these regions engage in certain kinds of protein-protein interactions that are essential for maintaining the general health of cells? It has been noted that ordered proteins engage in metabolism, catalysis etc while disordered regions are engaged in regulation and signalling^8^,^70^. In our search for mutations seen in cancers in this region, we have come across only one mutation, G398E. Earlier simulation studies^71^ on the binding of this mutant to the receptor S100B(ββ) suggested that it likely binds with higher affinity, thus leading to disabling of the transcription function of p53. A quick look at the electrostatic potential of two receptors of CTD, S100B(ββ) and CBP suggest that there must be some kinetic attenuation of the interactions with the former as a very anionic potential of S100B(ββ) is approached by a peptide now bearing a negative charge on G398E (Fig. S12) In contrast the cationic potential of CBP must enhance the uptake of the negatively charged mutant. If this mutation alleviates binding of p53 to S100B(ββ), then it certainly must enhance transcription as it would do if CBP bound the mutant with higher affinity^30^. It is clear that detailed experimental studies will be required to unpack the role of this promiscuous segment of p53.

## Additional Information

**How to cite this article**: Kannan, S. *et al.* Long range recognition and selection in IDPs: the interactions of the C-terminus of p53. *Sci. Rep.* **6**, 23750; doi: 10.1038/srep23750 (2016).

## Supplementary Material

Supplementary Information

## Figures and Tables

**Figure 1:**
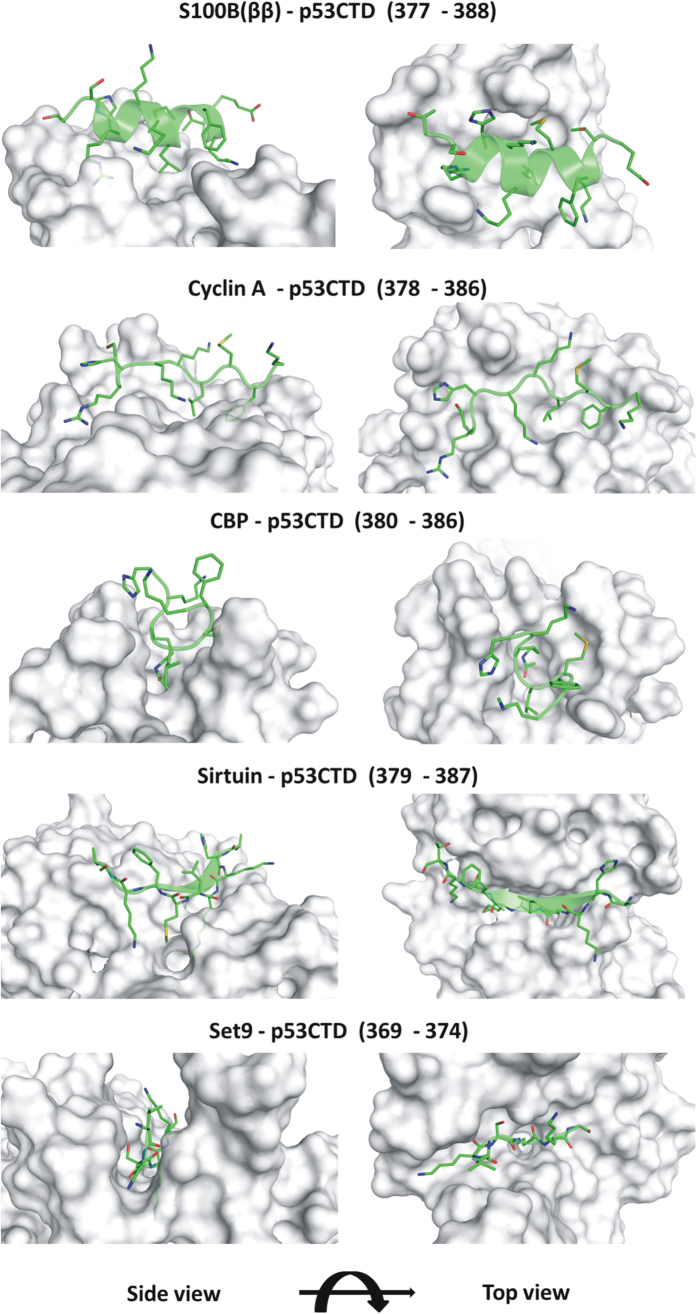
Conformations of p53CTD (Green) bound with different receptor proteins (Grey). p53CTD is shown as cartoon with side chain of peptide residues shown as sticks and the receptor protein shown as surface.

## References

[b1] Dunker A. K., Silman I., Uversky V. N. & Sussman J. L. Function and structure of inherently disordered proteins. Curr. Opin. Struct. Biol. 18, 756–764 (2008). 18952168 10.1016/j.sbi.2008.10.002

[b2] Dyson H. J. & Wright P. E. Intrinsically unstructured proteins and their functions. Nat. Rev. Mol. Cell Biol. 6, 197–208 (2005). 15738986 10.1038/nrm1589

[b3] Tompa P. Unstructural biology coming of age. Curr. Opin. Struct. Biol. 21, 419–425 (2011). 21514142 10.1016/j.sbi.2011.03.012

[b4] Dunker A. K., Obradovic Z., Romero P., Garner E. C. & Brown C. J. Intrinsic protein disorder in complete genomes. Genome Inform. Ser. Workshop Genome Inform. 11, 161–171 (2000). 11700597

[b5] Wright P. E. & Dyson H. J. Linking folding and binding. Curr. Opin. Struct. Biol. 19, 31–38 (2009). 19157855 10.1016/j.sbi.2008.12.003 PMC2675572

[b6] Wang J. *et al.* Multi-scaled explorations of binding-induced folding of intrinsically disordered protein inhibitor IA3 to its target enzyme. PLoS Comput. Biol. 7(4), e1001118 (2011). 21490720 10.1371/journal.pcbi.1001118 PMC3072359

[b7] Tompa P. Intrinsically unstructured proteins. TIBS. 27, 527–533 (2002). 12368089 10.1016/s0968-0004(02)02169-2

[b8] Wright P. E. & Dyson H. J. Intrinsically disordered proteins in cellular signalling and regulation. Nat. Rev. Mol. Cell. Biol. 16, 18–29 (2015). 25531225 10.1038/nrm3920 PMC4405151

[b9] Das R. K., Mao A. H. & Pappu R. V. Unmasking functional motifs within disordered regions of proteins. *Sci*. Signal. 5, pe17 (2012). 10.1126/scisignal.2003091 22510467

[b10] Davey N. E. *et al.* Attributes of short linear motifs. Mol. Biosyst. 8, 268–281 (2012). 21909575 10.1039/c1mb05231d

[b11] Espinoza-Fonseca L. M. Reconciling binding mechanisms of intrinsically disordered proteins. Biochem. Biophys. Res. Commun. 382, 479–482 (2009). 19265676 10.1016/j.bbrc.2009.02.151

[b12] Dogan J., Gianni S. & Jemth P. The binding mechanisms of intrinsically disordered proteins. Phys. Chem. Chem. Phys. 16, 6323–6331 (2014). 24317797 10.1039/c3cp54226b

[b13] Dyson H. J. & Wright P. E. Coupling of folding and binding for unstructured proteins Curr. Opin. Struct. Biol. 12, 54–60 (2002). 11839490 10.1016/s0959-440x(02)00289-0

[b14] Sugase K., Dyson H. J. & Wright P. E. Mechanism of coupled folding and binding of an intrinsically disordered protein. Nature. 447, 1021–1025 (2007). 17522630 10.1038/nature05858

[b15] Uversky V. N. Intrinsically disordered proteins from A to Z. Int. J. Biochem. Cell. Biol. 43, 1090–1103 (2011). 21501695 10.1016/j.biocel.2011.04.001

[b16] Kumar S., Ma B., Tsai C. J., Sinha N. & Nussinov R. Folding and binding cascades: dynamic landscapes and population shifts. Protein Sci. 9, 10–19 (2000). 10739242 10.1110/ps.9.1.10 PMC2144430

[b17] Tsai C. J., Ma B., Sham Y. Y., Kumar S. & Nussinov R. Structured disorder and conformational selection. Proteins. 44, 418–427 (2001). 11484219 10.1002/prot.1107

[b18] Uversky V. N., Oldfield C. J. & Dunker A. K. Intrinsically disordered proteins in human diseases: Introducing the D-2 concept. Annu. Rev. Biophys. 37, 215–246 (2008). 18573080 10.1146/annurev.biophys.37.032807.125924

[b19] Metallo S. J. Intrinsically disordered proteins are potential drug targets. Curr. Opin. Chem. Biol. 14, 481–488 (2010). 20598937 10.1016/j.cbpa.2010.06.169 PMC2918680

[b20] Heller G. T., Sormanni P. & Vendruscolo M. Targeting disordered proteins with small molecules using entropy. TIBS. 40, 491–496 (2015). 26275458 10.1016/j.tibs.2015.07.004

[b21] Zhang Z. *et al.* Chemical perturbation of an intrinsically disordered region of TIFD distinguishes two modes of transcription initiation. eLife. 10.7554/eLife.07777 (2015). PMC4582147 26314865

[b22] Lane D. P. p53, guardian of the genome. Nature. 358, 15–16 (1992). 1614522 10.1038/358015a0

[b23] Huart A. S. & Hupp T. R. Evolution of Conformational Disorder & Diversity of the P53 Interactome. BioDiscovery. 8, 5 (2013).

[b24] McKinney K., Mattia M., Gottifredi V. & Prives C. p53 Linear Diffusion along DNA Requires Its C Terminus. Mol. Cell. 16, 413–424 (2004). 15525514 10.1016/j.molcel.2004.09.032

[b25] Luo J. *et al.* Acetylation of p53 Augments its Site-Specific DNA Binding Both *in vitro* and *in vivo*. PNAS. 101, 2259– 2264 (2004). 14982997 10.1073/pnas.0308762101 PMC356938

[b26] Friedler A., Veprintsev D. B., Freund S. M. V., von Glos K. I. & Fersht A. R. Modulation of Binding of DNA to the C-Terminal Domain of p53 by Acetylation. Structure. 13, 629–636 (2005). 15837201 10.1016/j.str.2005.01.020

[b27] Freed-Pastor W. A. & Prives C. Mutant p53: one name, many proteins. Genes Dev. 26, 1268–1286 (2012). 22713868 10.1101/gad.190678.112 PMC3387655

[b28] Rustandi R. R., Baldisseri D. M. & Weber D. J. Structure of the negative regulatory domain of p53 bound to S100B(betabeta). Nat. Struct. Biol. 7, 570–574 (2000). 10876243 10.1038/76797

[b29] Valos J. L. *et al.* Structure of a Sir2 enzyme bound to an acetylated p53 peptide. Mol. Cell. 10, 523–535 (2002). 12408821 10.1016/s1097-2765(02)00628-7

[b30] Mujtaba S. *et al.* Structural mechanism of the bromodomain of the coactivator CBP in p53 transcriptional activation. Mol. Cell. 13, 251–263 (2004). 14759370 10.1016/s1097-2765(03)00528-8

[b31] Chuikov S. *et al.* Regulation of p53 activity through lysine methylation. Nature. 18, 353–360 (2004). 15525938 10.1038/nature03117

[b32] Lowe E. D. *et al.* Specificity determinants of recruitment peptides bound to phospho-CDK2/cyclin A. Biochemistry. 41, 15625–15634 (2002). 12501191 10.1021/bi0268910

[b33] McDowell C., Chen J. & Chen J. Potential Conformational Heterogeneity of p53 bound to S100B(betabeta). J. Mol. Biol. 425, 999–1010 (2013). 23313430 10.1016/j.jmb.2013.01.001

[b34] Chen J. Intrinsically Disordered p53 Extreme C-Terminus Binds to S100B(ββ) through Fly-Casting. J. Am. Chem. Soc. 131, 2088– 2089 (2009). 19216110 10.1021/ja809547p

[b35] Allen W. J., Capelluto D. G., Finkielstein C. V. & Bevan D. R. Modeling the relationship between the p53 C-terminal domain and its binding partners using molecular dynamics. J. Phys. Chem. B. 114, 13201–13213 (2010). 20873738 10.1021/jp1011445

[b36] Staneva I., Huang Y., Liu Z. & Wallin S. Binding of Two Intrinsically Disordered Peptides to a Multi-Specific Protein: A Combined Monte Carlo and Molecular Dynamics Study. PLoS. Comput. Biol. 8(9), e1002682 (2012). 23028280 10.1371/journal.pcbi.1002682 PMC3441455

[b37] Case D. *et al.* Amber 11. (University of California, San Francisco, 2010).

[b38] Jorgensen W. L., Chandrasekhar J., Madura J. D., Impey R. W. & Klein M. L. Comparison of simple potential functions for simulating liquid water. J. Chem. Phys. 79, 926–935 (1983).

[b39] Duan Y. *et al.* Pointcharge force field for molecular mechanics simulations of proteins based on condensed-phase quantum mechanical calculations. J. Comput. Chem. 24, 1999–2012 (2003). 14531054 10.1002/jcc.10349

[b40] Homeyer N., Horn A. H. C., Lanig H. & Sticht H. AMBER force field parameters for phosphorylated amino acids in different protonation states: phosphoserine, phosphothreonine, phosphotyrosine and phosphohistidine. J. Mol. Model. 12, 281–289 (2006). 16240095 10.1007/s00894-005-0028-4

[b41] Isralewitz B., Baudry J., Gullingsrud J., Kosztin D. & Schulten K. Steered molecular dynamics investigations of protein function. J. Mol. Graph. Model. 19, 13–25 (2001). 11381523 10.1016/s1093-3263(00)00133-9

[b42] Marco E. & Gago F. Overcoming the inadequacies or limitations of experimental structures as drug targets by using computational modeling tools and molecular dynamics simulations. ChemMedChem. 2, 1388–1401 (2007). 17806089 10.1002/cmdc.200700087

[b43] Gua Y., Shrivastava I. H., Amara S. G. & Bahar I. Molecular simulations elucidate the substrate translocation pathway in a glutamate transporter. PNAS. 106, 2589–2594 (2009). 19202063 10.1073/pnas.0812299106 PMC2637273

[b44] Martinez L., Polikarpov I. & Skaf M. S. Only subtle protein conformational adaptations are required for ligand binding to thyroid hormone receptors: Simulations using a novel multipoint steered molecular dynamics approach. J. Phys. Chem. B. 112, 10741–10751 (2008). 18681473 10.1021/jp803403c

[b45] Sonoda M. T., Martinez L., Webb P., Skaf M. S. & Polikarpov I. Ligand dissociation from estrogen receptor Is mediated by receptor dimerization: evidence from molecular dynamics simulations. Mol. Endocrinol. 22, 1565–1578 (2008). 18403716 10.1210/me.2007-0501 PMC5419439

[b46] Phillips J. C. *et al.* Scalable molecular dynamics with NAMD. J. Comput. Chem. 26, 1781–1802 (2005). 16222654 10.1002/jcc.20289 PMC2486339

[b47] Homeyer N. & Gohlke H. Free Energy Calculations by the Molecular Mechanics Poisson-Boltzmann Surface Area Method. Mol. Inf. 31, 114–122 (2012). 10.1002/minf.201100135 27476956

[b48] Feig M., Karanicolas J. & Brooks C. L. MMTSB tool set: enhanced sampling and multiscale modeling methods for applications in structural biology. J. Mol. Graph. Model. 22, 377–395 (2004). 15099834 10.1016/j.jmgm.2003.12.005

[b49] Humphrey W., Dalke A. & Schulten K. VMD—visual molecular dynamics. J. Mol. Graph. 14, 33–38 (1996). 8744570 10.1016/0263-7855(96)00018-5

[b50] De Lano W. The PyMOL molecular graphics system, version 1.3, Schrodinger, LLC. URL: https://www.pymol.org.

[b51] Shoemaker B. A., Portman J. J. & Wolynes P. G. Speeding molecular recognition by using the folding funnel: the fly-casting mechanism. PNAS. 97, 8868–8873 (2000). 10908673 10.1073/pnas.160259697 PMC16787

[b52] Fuxreiter M., Simon I., Friedrich P. & Tompa P. Preformed structural elements feature in partner recognition by intrinsically unstructured proteins. J. Mol. Biol. 338, 1015–1026 (2004). 15111064 10.1016/j.jmb.2004.03.017

[b53] Receveur-Bréchot V., Bourhis J. M., Uversky V. N., Canard B. & Longhi S. Assessing Protein Disorder and Induced Folding. Proteins. 62, 24–45 (2006). 16287116 10.1002/prot.20750

[b54] Bienkiewicz E. A., Adkins J. N. & Lumb K. J. Functional consequences of preorganized helical structure in the intrinsically disordered cell-cycle inhibitor p27(Kip1). Biochemistry 41, 752–759 (2002). 11790096 10.1021/bi015763t

[b55] Moritsugu K., Terada T. & Kidera A. Disorder-to-order transition of an intrinsically disordered region of sortase revealed by multiscale enhanced sampling. J. Am. Chem. Soc. 134, 7094–7101 (2012). 22468560 10.1021/ja3008402

[b56] Tran H. T., Mao A. & Pappu R. V. Role of backbone-solvent interactions in determining conformational equilibria of intrinsically disordered proteins. J. Am. Chem. Soc. 130, 7380–7392 (2008). 18481860 10.1021/ja710446s

[b57] Huang Y. & Liu Z. Kinetic advantage of intrinsically disordered proteins in coupled folding-binding process: a critical assessment of the “fly-casting” mechanism. J. Mol. Biol. 393, 1143–1159 (2009). 19747922 10.1016/j.jmb.2009.09.010

[b58] Brocca S. *et al.* Compaction Properties of an Intrinsically Disordered Protein: Sic1 and Its Kinase-Inhibitor Domain. Biophys. J. 100, 2243–2252 (2011). 21539793 10.1016/j.bpj.2011.02.055 PMC3149264

[b59] Teufel D. P., Johnson C. M., Lum J. K. & Neuweiler H. Backbone-Driven Collapse in Unfolded Protein Chains. J. Mol. Biol. 409, 250–262 (2011). 21497607 10.1016/j.jmb.2011.03.066

[b60] Papadakos G. *et al.* Consequences of Inducing Intrinsic Disorder in a High-Affinity Protein–Protein Interaction. J. Am. Chem. Soc. 137, 5252–5255 (2015). 25856265 10.1021/ja512607r

[b61] Okazaki K. & Takada S. Dynamic energy landscape view of coupled binding and protein conformational change: induced-fit versus population-shift mechanisms. PNAS. 105, 11182–11187 (2008). 18678900 10.1073/pnas.0802524105 PMC2516237

[b62] Arai M., Sugase K., Dyson H. J. & Wright P. E. Conformational propensities of intrinsically disordered proteins influence the mechanism of binding and folding. PNAS. 112, 9614–9619 (2015). 26195786 10.1073/pnas.1512799112 PMC4534220

[b63] Zhang W., Ganguly D. & Chen J. Residual Structures, Conformational Fluctuations, and Electrostatic Interactions in the Synergistic Folding of Two Intrinsically Disordered Proteins. PLoS Comput. Biol. 8(1), e1002353 (2012). 22253588 10.1371/journal.pcbi.1002353 PMC3257294

[b64] Ganguly D. & Chen J. Atomistic details of the disordered states of KID and pKID. Implications in coupled binding and folding. J. Am. Chem. Soc. 131, 5214–5223 (2009). 19278259 10.1021/ja808999m

[b65] Kurcinski M., Kolinski A. & Kmiecik S. Mechanism of folding and binding of an intrinsically disordered protein as revealed by ab initio simulations. J. Chem. Theory. Comput. 10, 2224–2231 (2014). 26580746 10.1021/ct500287c

[b66] Petitjean A. *et al.* Impact of mutant p53 functional properties on TP53 mutation patterns and tumor phenotype: lessons from recent developments in the IARC TP53 database. Hum. Mutat. 28, 622–629 (2007). 17311302 10.1002/humu.20495

[b67] Forbes S. A. *et al.* COSMIC: exploring the world’s knowledge of somatic mutations in human cancer. Nucl. Acids Res. 43, D805–D811 (2015). 25355519 10.1093/nar/gku1075 PMC4383913

[b68] Malaney P., Pathak R. R., Xue B., Uversky V. N. & Dave V. Intrinsic Disorder in PTEN and its Interactome Confers Structural Plasticity and Functional Versatility. Sci. Rep. 3, 2035 (2013). 23783762 10.1038/srep02035 PMC3687229

[b69] Vacic V. & Iakoucheva L. M. Disease mutations in disordered regions—exception to the rule? Mol. Biosyst. 8, 27–32 (2012). 22080206 10.1039/c1mb05251a PMC3307532

[b70] Teilum K., Olsen J. G. & Kragelund B. B. Globular and disordered—the non-identical twins in protein-protein interactions. Front. mol. biosci. 2, 40 (2015). 26217672 10.3389/fmolb.2015.00040 PMC4496568

[b71] Pirolli D. *et al.* Insight into a novel p53 single point mutation (G389E) by Molecular Dynamics simulations. Int. J. Mol. Sci. 12, 128–140 (2011). 21339981 10.3390/ijms12010128 PMC3039947

