# Supplementary material for: Long range recognition and selection in IDPs: the interactions of the C-terminus of p53
Source: Sci Rep. 2016 Mar 31;6:23750. doi: 10.1038/srep23750 (PMC4814905; doi:10.1038/srep23750)
Supplement: Supplementary Information [file srep23750-s1.doc]

**Supplementary Information**

**Long range recognition and selection in IDPs: the interactions of the C-terminus of p53**

***Srinivasaraghavan Kannan,^a*^ David P Lane,^b^ Chandra S. Verma,^a,c,d*^***

*^a^*Bioinformatics Institute (A*STAR), 30 Biopolis Street, #07-01 Matrix, Singapore 138671 ^b^p53 Laboratory (A*STAR), 8A Biomedical Grove, #06-04/05, Neuros/Immunos, Singapore 138648

*^c^*School of Biological Sciences, Nanyang Technological University, 60 Nanyang Drive,

Singapore 637551

*^d^*Department of Biological Sciences, National University of Singapore, 14 Science Drive 4, Singapore 117543

**Tables**

**Table S1A:** Details of standard MD simulations. All systems were simulated in triplicates and 100ns each.

| **System** | **State** | **Peptide length** |
| --- | --- | --- |
| S100B(ββ) – p53CTD | Complex | 12 residues (377 to 388) |
|  | Apo p53CTD | 12 residues (377 to 388) |
|  | Apo S100B(ββ) Monomer |  |
| Cyclin A – p53CTD | Complex | 9 residues (378 to 386) |
|  | Apo p53CTD | 9 residues (378 to 386) |
|  | Apo Cyclin A |  |
| CBP bromodomain – p53CTD | Complex | 7 residues (380 to 386) |
|  | Apo p53CTD | 7 residues (380 to 386) |
|  | Apo CBP bromodomain |  |
| Sirtuin – p53CTD | Complex | 9 residues (379 to 387) |
|  | Apo p53CTD | 9 residues (379 to 387) |
|  | Apo Sirtuin |  |
| Set9 – p53CTD | Complex | 6 residues (369 to 374) |
|  | Apo p53CTD | 6 residue (369 to 374) |
|  | Apo Set9 |  |

**Table S1B:** Details of p53CTD folding MD simulations.

| **System** | **State** | **Types of simulations** | **Timescale** |
| --- | --- | --- | --- |
| p53CTD –  20 residues (370 to 389) | Extended | Standard MD | 200ns x 5 |
|  |  | T-REMD with 24 replicas (temp) | 50ns x 24 |
|  |  | BP-REMD with 5 replicas | 50ns x 5 |

**Table S1C:** Details of p53CTD unbinding SMD simulations.

| **System** | **No of simulations** | **Timescale** |
| --- | --- | --- |
| S100B(ββ) – p53CTD | 5 | 10ns each |
| Cyclin A – p53CTD | 5 | 10ns each |
| CBP bromodomain – p53CTD | 5 | 10ns each |
| Sirtuin – p53CTD | 5 | 10ns each |
| Set9 – p53CTD | 5 | 10ns each |

**Table S1D:** Details of umbrella sampling simulations.

| **System** | **Peptide position** | **Peptide conformation** | **Timescale** |
| --- | --- | --- | --- |
| S100B(ββ) – p53CTD | At binding site | Alpha, beta, disordered | 25ns each |
|  | 10Å from binding site | Alpha, beta, disordered | 25ns each |
|  | 20Å from binding site | Alpha, beta, disordered | 25ns each |
|  | 30Å from binding site | Alpha, beta, disordered | 25ns each |
| Cyclin A – p53CTD | At binding site | Alpha, beta, disordered | 25ns each |
|  | 10Å from binding site | Alpha, beta, disordered | 25ns each |
|  | 20Å from binding site | Alpha, beta, disordered | 25ns each |
|  | 30Å from binding site | Alpha, beta, disordered | 25ns each |
| Sirtuin – p53CTD | At binding site | Alpha, beta, disordered | 25ns each |
|  | 10Å from binding site | Alpha, beta, disordered | 25ns each |
|  | 20Å from binding site | Alpha, beta, disordered | 25ns each |
|  | 30Å from binding site | Alpha, beta, disordered | 25ns each |


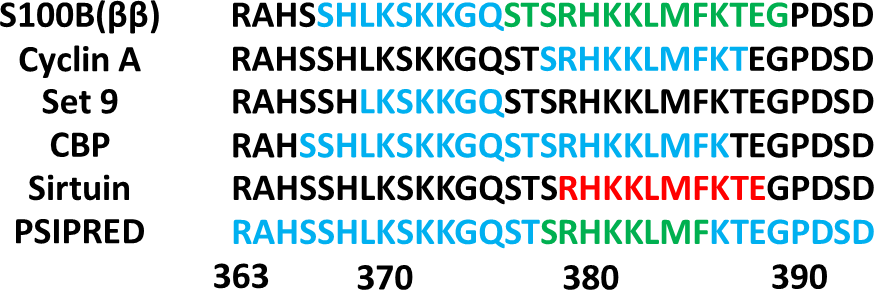


**Figure S1:** CTD of p53 that binds with various partners. The experimentally solved structures of the bound regions are coloured according to the structural property Green: alpha helix, Red: Beta – Sheet, Blue: disordered and or turn conformation. The last line (PSIPRED) corresponds to the secondary structure predicted using PSIRPED [Buchan DWA, Minneci F, Nugent TCO, Bryson K, Jones DT. (2013). *Nucleic Acids Research.*  41 (W1): W340-W348.] server.

**p53 folding simulations**


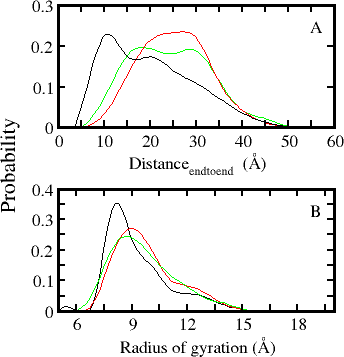


**Figure S2:** Probability distribution of end to end distance (A) and Rg values (B) of disordered ensemble of free p53 extreme C-terminus from the fully extended state during sMD (black), BP-REMD (red) T-REMD (green) simulations.

For the p53CTD folding simulations (simulation initiated from a fully extended state) 5 independent MD simulations were carried out for 200ns each by assigning different initial velocities (Table S1B).  In addition to sMD, the linear peptide fragment was subjected to REMD simulations to enhance the conformational sampling. Two different types of replica exchange methods were used: 1) a standard temperature-based T-REMD^1^ was carried out under constant volume using 24 replicas. An exponentially increasing temperature series along the replicas was used which gives approximately uniform acceptance ratios for exchanges between neighbouring replicas with temperature ranges  from 300.0 K to 400.5 K These simulation temperatures resulted in exchange probabilities between neighboring replicas of ~25%. The T-REMD simulations were continued for 50ns. 2) A biasing-potential REMD^2^ that is a Hamiltonian based REMD (BP-REMD) with conformational sampling enhanced using a specifically designed dihedral potential^2^. In a BP-REMD simulation, different biasing potential levels are applied in each replica (one reference replica run is carried out without any biasing potential) and replica exchanges between neighboring biasing levels were attempted every 1000 MD steps and accepted or rejected according to a Metropolis criterion. The BP–REMD simulations were carried out at 300 K with 5 replicas with the same biasing potential and biasing levels as given in Table I of reference^2^ and the simulations were extended to 50ns. The acceptance probability for replica exchanges was in the range of 30-40%.

We examined the conformational landscape of a 20 residue segment/fragment of the p53CTD (residues 370-389) after initiating the simulations in a modeled extended state. During the simulations, the isolated p53CTD samples compact and extended states (rg varies between 6.5Å and 15Å, peaking around 8-10Å; Figure S2). The free peptide tends to adopt mostly collapsed states (end to end distances ~10Å, Figure S2) during sMD and extended states (end to end distance >20Å, Figure S2) during both REMD. The calculated residual helicity suggests that approximately 50% of the sampled conformations are helical, especially the region spanned by residues 375-389. This region (residues 376-390) adopts an alpha helical conformation when bound to S100B(ββ) (Figure 1, S1). A significant population of extended helical conformations (residues 370-389) was also sampled during both the REMD simulations (Figure S5, S6). The centroids of the 5 most populated clusters of conformations sampled during the REMD simulations represent alpha helical and extended alpha helical states (Figure S6). In contrast, most of the conformations sampled by free p53CTD during sMD adopt a beta-turn-beta motif that is stabilized by intra molecular interactions between two beta sheets. Small populations of helical conformations (residues 370- 389) were seen in sMD, likely due to kinetic trapping (Figure S5, S6). In addition there is a substantial probability for the peptide to adopt turn conformations especially at the N-terminus (residues 370-377) of p53CTD (Figure S5); this region (residues 369-375) adopts a turn when bound with Set9 (Figure 1, S1).  p53CTD  (residues 379-388) adopts a beta sheet conformation upon binding to Sirtuin (Figure 1, S1), but this conformation was sampled with very low probability.  This is understandable since for a segment to adopt a beta strand, it needs other strand-like templates in its vicinity to be stabilized by inter-chain backbone h bonds, which are missing in the apo state.

To examine the existence of bound conformations in the unbound ensemble of p53CTD we calculated the pair-wise rmsd between the apo (obtained from the sMD, T-REMD and BP-REMD) from each of these folded conformations. It is clear (Figure S3) that there are significant populations of conformational states sampled in the unbound states that are very similar to the folded complexed states (pair wise rmsd < ~3Å). The percentage of folded/bound conformations sampled in the apo simulations started from the extended state was estimated (Figure S4). The disordered or beta turn bound conformation of p53CTD was sampled with high probability. In contrast, the alpha helical and beta sheet bound conformations were sampled with varying probabilities depending on the methods used for the simulations. BP-REMD samples the alpha helical state at ~50% but the beta sheet only at ~20%. The T-REMD also samples the beta sheet conformation at ~20%, but the alpha helical conformation is sampled less than 20%. Conventional MD simulations sample the beta sheet bound conformation at ~25% but the alpha helical conformation is sampled at only ~10%.


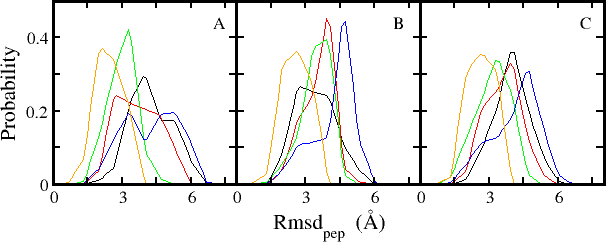


**Figure S3:** Probability distribution of rmsd values of disordered ensemble of free p53 extreme C-terminus during sMD (A), BP-REMD (B) T-REMD (C) simulations starting from its fully extended state against the five bound conformations. Only the folded/bound segments were included in the calculation. The disordered ensemble includes all the conformation sampled either during the five independent standard simulations in the case of sMD (A) at the reference replica with the original force filed in the case of BP-REMD (B) at the lowest temperature during the T-REMD. Colours represent different bound (folded) conformation of the p53CTD bound with different receptor protein. Black, red, green, blue and orange corresponds to the conformation of p53CTD bound with S100b(ββ) (alpha helix) , Cyclin A (disordered), CBP (disordered), Sirtuin (beta sheet) and Set9 proteins (turn).


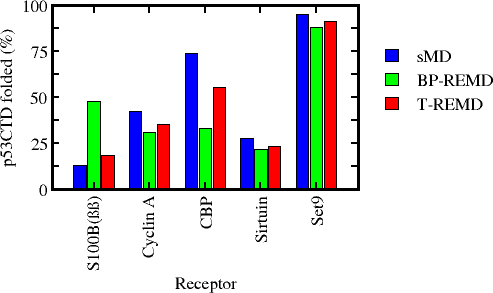


**Figure S4:** Percentage of bound (folded) conformations from the disordered ensemble of free p53 extreme C-terminus from the five bound(folded) conformations during sMD (blue), BP-REMD (green) T-REMD (red) simulations starting from its fully extended state. Only the bound (folded) segments were included in the calculation and cut-off of 3Å was used to quantify the bound (folded) state. The disordered ensemble includes all the conformation sampled either during the five independent continuous simulations in the case of sMD (blue) at the reference replica with the original force filed in the case of BP-REMD (green) at the lowest temperature during the T-REMD (red).


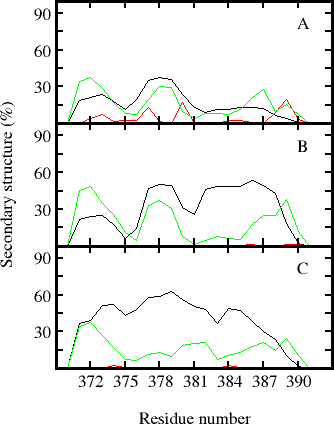


**Figure S5:** Percentage of secondary structures (Black: alpha helix, Red: beta sheet, Green: turn) sampled in the disordered ensemble of free p53 extreme C-terminus from the fully extended state during sMD (A), BP-REMD (B) T-REMD (C) simulations.


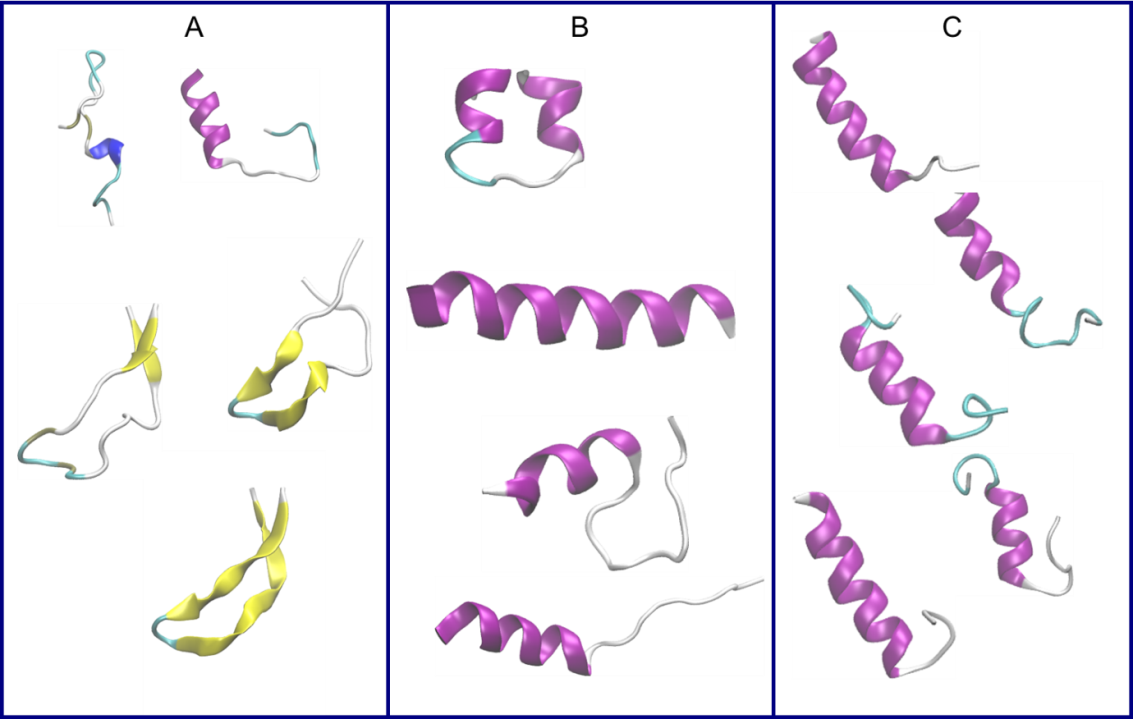


**Figure S6:** Cluster centroids of highly populated clusters from the disordered ensemble of free p53 extreme C-terminus during sMD (A), BP-REMD (B) T-REMD (C) simulations starting from its fully extended state. The disordered ensemble includes all the conformation sampled either during the five independent continuous simulations in the case of sMD (A) at the reference replica with the original force filed in the case of BP-REMD (B) at the lowest temperature during the T-REMD (C).


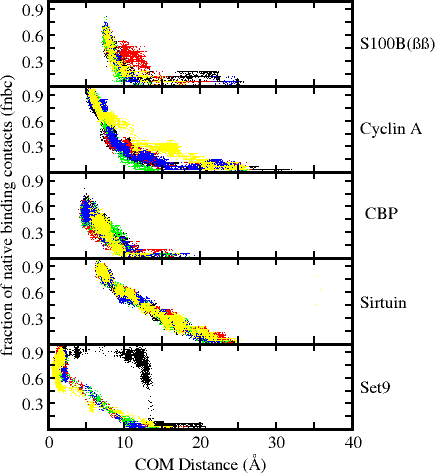


**Figure S7:** Fractions of native binding contacts (fnbc) formed between the receptor – p53CTD peptide fragment as a function of centre of mass distance (COM) between the peptide – receptor complex. All the conformations sampled during SMD (5 independent for each system) are included here highlighted in different colours.


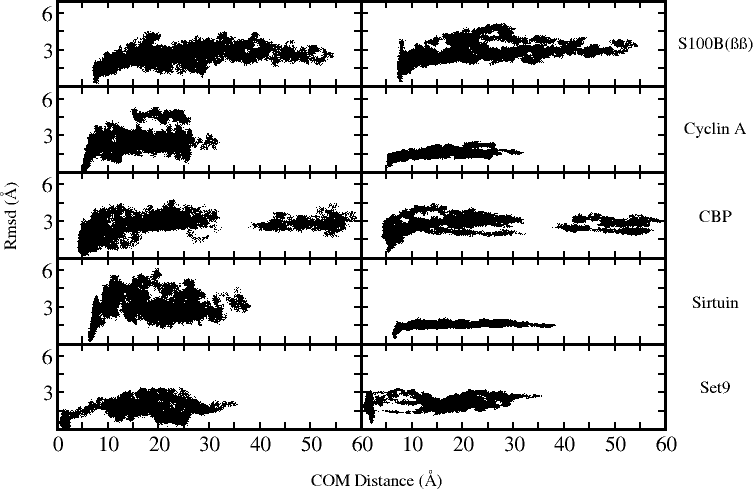


**Figure S8:** RMSD of bound p53CTD peptide (right) and corresponding receptor (left) as a function of centre of mass distance (COM) between the peptide and receptor from its complex. All the conformations sampled during SMD (5 independent for each system) are included here.


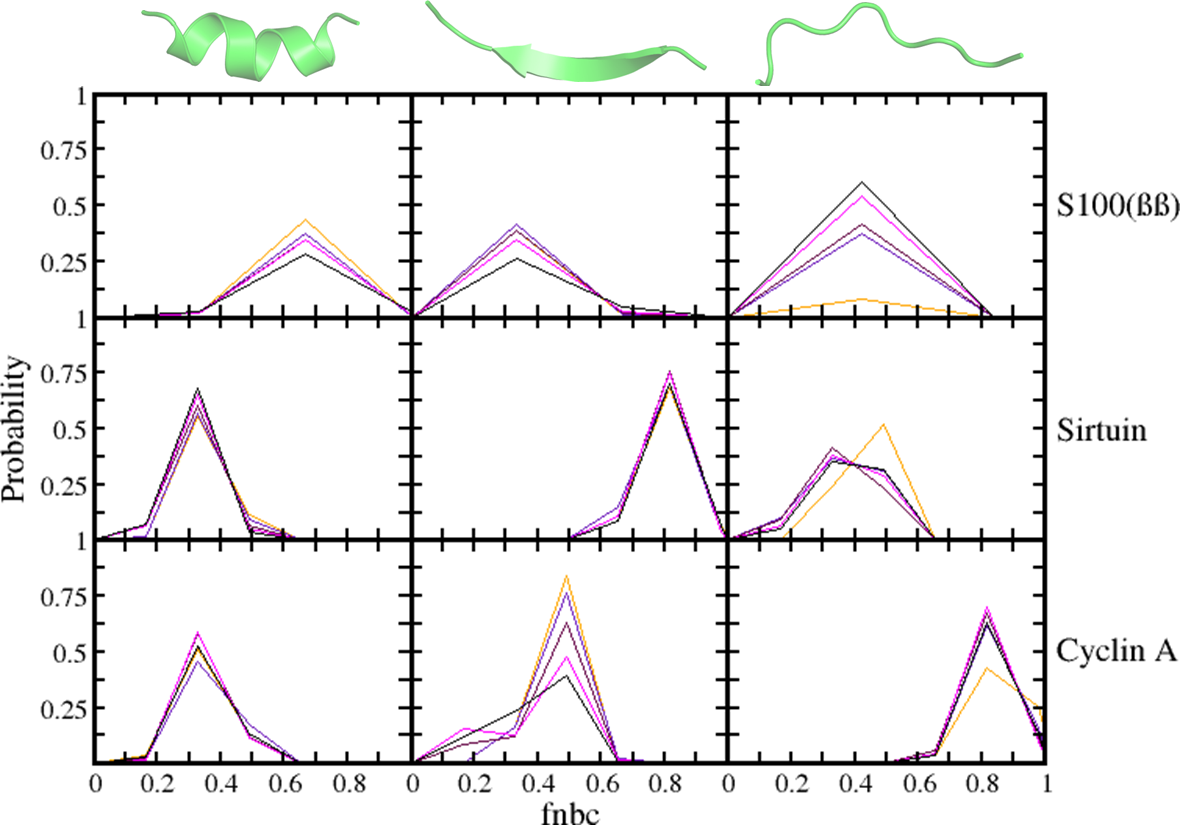


**Figure S9:** Distribution of fractions of native binding contacts (fnbc) formed between the receptor and p53CTD peptide fragment at the binding site during the 25ns US MD simulations. Different colours correspond to fnbc calculated during different time period: Orange: 0 to 5ns, Indigo: 0 to 10ns Maroon: 0 to 15ns, Magenta: 0 to 20ns and Black: 0 to 25ns. And the significant overlap between the fnbc at the different time periods shows the convergence. All the conformations sampled during US MD simulations with bound form of p53CTD fragment either in its native or non –native conformations bound with three receptor protein at the binding site are included.


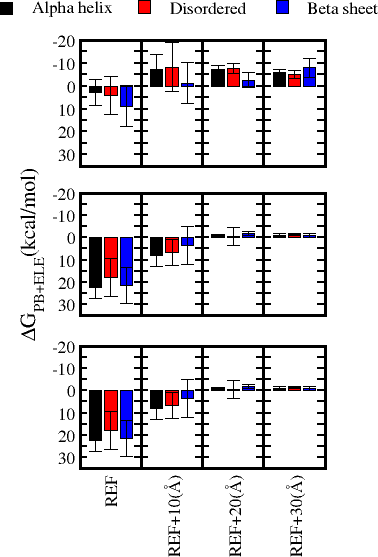


**Figure S10:** Contribution of desolvation to the total binding free energy of three p53CTD peptide – receptor complexes. All the conformations sampled during US MD simulations with bound form p53CTD fragment either in its native and non –native conformations at the binding site and various distances from the binding site are included in the binding energy calculations.


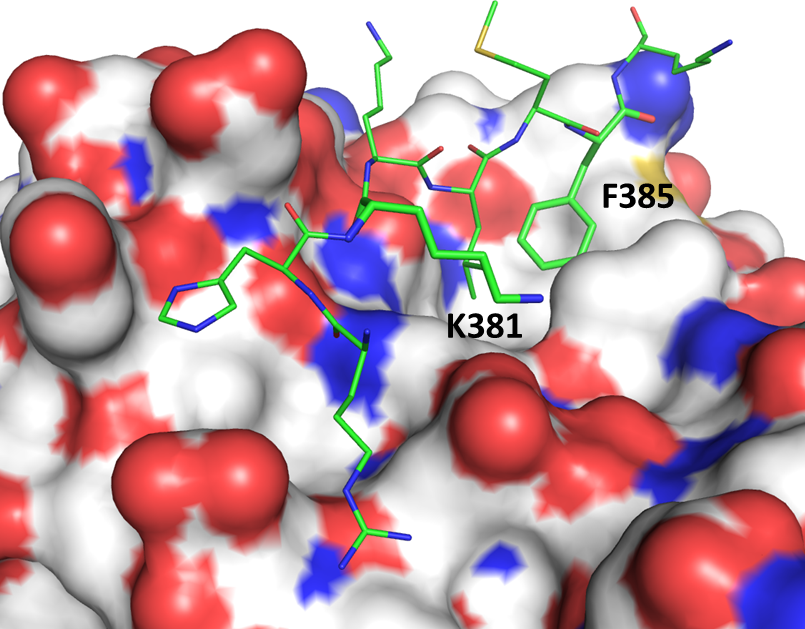


**Figure S11:** Conformation of p53CTD (Green) bound with Cyclin A. p53CTD is shown as lines with two residues (K381, F385) mutated to alanine are highlighted (thick lines). Cyclin A receptor is shown as surface (atomic colour).


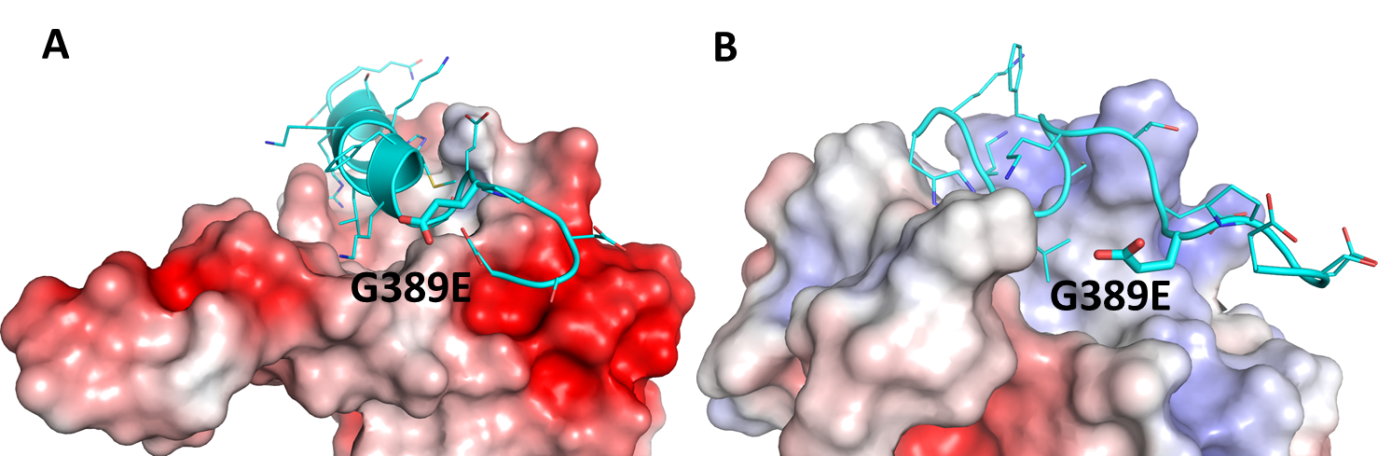


**Figure S12:** Conformation of p53CTD (Cyan) bound with (A) S100B(ββ) and Cyclin A (B). p53CTD is shown as cartoon with residue (G389) mutated to Glutamic acid (G389E) is highlighted (thick lines). S100B(ββ) and Cyclin A receptor is shown as surface (electrostatics).

**References:**

1. Sugita, Y.; Okamoto, Y. Replica-exchange molecular dynamics method for protein folding. *Chem. Phys. Lett.* **1999,**  314, 141-151.
2. Kannan, S.; Zacharias, M. Enhanced sampling of peptide and protein conformations using replica exchange simulations with a peptide backbone biasing-potential. *Proteins.* **2007,** 66, 697-706.
